# The Book World of Medicine and Science

**Published:** 1899-02-11

**Authors:** 


### THE BOOK WORLD OF MEDICINE AND SCIENCE.

THE TREATMENT OF DISEASE BY PHYSICAL METHODS. By THOMAS STRETCH DOWSE, M.D., F.R.C.P.Ed. (Bristol: John Wright and Co. 1898. Price 7s. 6d. net.)

IN issuing a new edition of his book Dr. Stretch Dowse has done well to replace its old title, namely, "Lectures on Massage and Electricity in the Treatment of Disease," by one which can properly cover all the "physical methods" of treatment which have of late years been introduced. Massage still remains the principal staple of the book, the main theme on which the author descants with the greatest authority; and as a guide to this art, or method, or whatever we choose to call it, the book is not only easy reading, but contains a good deal which many of its readers will find both interesting and instructive. The first difficulty we meet with in reviewing such a book is that we do not quite see to whom it is addressed. If addressed to medical men much of it is evidently redundant; while, if to the lay public, not only is it redundant, but we fear that a good deal may be either incomprehensible or, indeed, absolutely misleading. But to the medical man who is not familiar with the physical methods now in vogue in the treatment of disease, and who does not mind skipping a good deal, the book will be found useful enough. It seems, however, that it is to the would-be masseur that it is really addressed, and this leads us to wonder whether for the practice of massage it is necessary to know all that is so familiarly discussed in this treatise. If so, it is clear that to be properly performed it should be done only by medical practitioners. Fortunately for our patients a good deal of most useful massage is done every day by people whose ignorance of physiology is only equalled by the skilfulness of their finger tips. After an introduction on the principles of massage and a description of the methods of its application, we have separate chapters on its use in different parts of the body and for the treatment of different diseases, interspersed with others on the Weir-Mitchell treatment, on Faradic massage, and on the Schott treatment, and we finish up with two lectures on electro-physics and electro-therapeutics. It would seem as if in some cases the boundary line between massage and hypnotism is but slight, and when we read of what is required in performing massage with the object of inducing sleep we may question whether the line is not sometimes overpassed. "It requires and demands a special organisation, a lightness of touch, a sense of confidence, and an assurance of results which all do not possess. The character of the masseur must be imbued with motive, induction, persuasion, solicitation, encouragement, and inspiration; the power of control, restraint, and authority must be so exercised with purposiveness and intenseness that the patient is made aware of his influence and yields himself to his treatment with a spirit of obedience, subjection, and unswerving trust." This reads like something more than a "physical" method of treatment, unless we are to look on all mental processes as matters of physics. Having seen "that the feet are to the south, and the head to the north," and "having arranged everything for the absolute quiet, ease, and composure of the patient, and, as far as possible, having subordinated the will of the patient to that of the operator, the patient's eyes are closed, and the lightest possible effleurage movements are made over the forehead, temples, eyes, and behind the ears, chiefly in the direction of the nerves. (It must be remembered that under appropriate conditions it is quite possible to influence patients to sleep.) . . . It must not be forgotten that these manipulations, to be effective, must be slow, purposive, and impressive, so that in a few minutes the patient will be rendered somewhat unconscious." There are coarse-minded people, no doubt, who might feel inclined on reading these paragraphs to say "humbug." We, however, merely quote them to show that massage is in some cases a matter rather of psychology than of physics; in other words, that it really spells hypnotism. As to the book there is to be found in it much that is interesting. It is clear that it is written by one who, as he himself says, is "a bit of an enthusiast concerning the valuable effects of massage," but it is none the worse for that. One feels that, at least, one is getting the case for massage stated as fully as it can be, although it must be admitted that the attempts at scientific explanation of a treatment which after all is full of empiricism are often somewhat ponderous. As we close this treatise we cannot avoid feeling that Dr. Stretch Dowse might perhaps have done better if he had written two small books instead of one large one; one book, *i.e.*, for the medical profession, giving us the results of his experience, showing us the rationale of massage and indicating the forms of treatment required in different cases, and another for the masseur, telling him exactly what to do in applying the method to the patient. Both or either of these Dr. Dowse could have done well. To combine the two, however, is very difficult, and in trying to do it Dr. Dowse has been led to speak to the masseur, who we suppose is a non-medical man, in such a manner and upon such subjects as to seem to imply that he possesses a considerable smattering of medical knowledge; just such a smattering, in fact, as to make him a most objectionable person—which in truth he sometimes is.

HERBERT FRY'S ROYAL GUIDE TO THE LONDON CHARITIES FOR 1899. Edited by JOHN LANE. (London: Chatto and Windus. Price 1s. 6d.)

THE new edition of this well-known annual contains a large amount of useful information concerning the charities which have their headquarters in London. The arrangement of the book is good, and the information appears to be accurate and recent. We have no doubt it will prove very useful to those for whose information it has been compiled.

GOLDEN RULES OF SURGICAL PRACTICE. By HURRY FENWICK, F.R.C.S.

GOLDEN RULES OF GYNÆCOLOGY. By S. JERVOIS AARONS, M.D.

GOLDEN RULES OF OBSTETRIC PRACTICE. By W. E. FOTHERGILL, M.A., B.Sc., M.D. (Bristol: John Wright and Co. Price 1s. each.)

MR. HURRY FENWICK'S "Golden Rules of Surgical Practice" have attained a fifth edition, and now they form Part I. of a Golden Rules Series, which is likely, we think, to gain considerable popularity. They are pretty little books, gold lettered and bound in white, which will go into the waistcoat pocket. We are not quite clear as to the occasions on which they are to be read, but we can have no doubt that when read and remembered they will be found to give useful hints in many emergencies. On the whole, the gynæcology seems the weaker book, perhaps from its very positiveness; still, as a rule, the precepts given may be easily followed.

